# Effect of Receptor Structure and Length on the Wrapping of a Nanoparticle by a Lipid Membrane

**DOI:** 10.3390/ma7053855

**Published:** 2014-05-14

**Authors:** Haizhen Zhang, Ling Wang, Bing Yuan, Kai Yang, Yuqiang Ma

**Affiliations:** 1 Center for Soft Condensed Matter Physics and Interdisciplinary Research, Soochow University, Suzhou 215006, Jiangsu, China; E-Mails: 20114209102@suda.edu.cn (H.Z.); 20114208003@suda.edu.cn (L.W.); yuanbing@suda.edu.cn (B.Y.); 2 College of Chemistry, Chemical Engineering and Material Science, Soochow University, Suzhou 215123, Jaingsu, China; 3 College of Physics, Optoelectronics and Energy, Soochow University, Suzhou 215006, Jiangsu, China; 4 National Laboratory of Solid State Microstructures and Department of Physics, Nanjing University, Nanjing 210093, Jiangsu, China

**Keywords:** lipid membrane, nanoparticle, receptor, endocytosis, computer simulations

## Abstract

Nanoparticles have been considered as a type of powerful tool to deliver drugs and genes into cells for disease diagnosis and therapies. It has been generally accepted that the internalization of nanoparticles into cells is mostly realized by receptor-mediated endocytosis. However, for the influence of structural factors of receptors on endocytosis, this is still largely unknown. In this paper, computer simulations are applied to investigate the effects of structure (*i.e.*, the number of constituent chains of the receptor) and the length of the receptor on the wrapping behavior of nanoparticles by the lipid membrane, which is a key step of receptor-medicated endocytosis. It is found that these structural factors of receptors have strong effects on the nanoparticle’s final interaction configuration with the membrane in the simulations, such as adhering on the membrane surface or being partly or fully wrapped by the membrane. Furthermore, in some cases, the rupture of the lipid membrane occurs. These results are helpful for the understanding of endocytosis and the preparation of advanced nanoscale drug-delivery vectors.

## 1. Introduction

Advances in nanotechnology greatly provoke the development of biomedicines [1–3]. Especially, nanoparticles have been considered as one of the powerful tools to deliver drugs and genes into cells [3,4]. Furthermore, they are proper candidates for cell imaging [5]. These applications are undoubtedly helpful for the diagnosis and treatment of diseases on the cell level [6].

**Figure 1.**
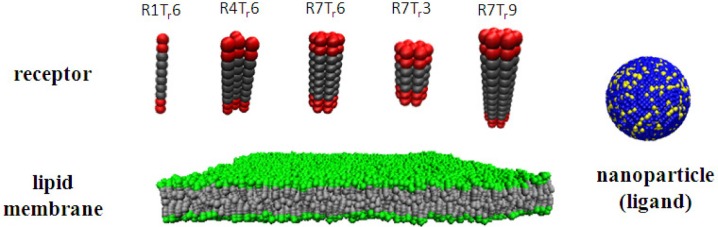
Sketch of the receptor, lipid membrane and nanoparticle (with ligands) used in the simulations. The receptor is named R(*N_m_*)T_r_(*N_t_*), in which *N_m_* stands for the number of polymer chains composing the receptor and *N_t_* is the number of hydrophobic beads in each chain. Red: active beads at the two ends of a receptor (*i.e.*, interaction sites with ligands, H_r_); dark grey: beads composing the backbone of a receptor chain (T_r_); yellow: ligands on a nanoparticle surface (L); blue: nanoparticle (P); green: lipid heads (H); grey: lipid tails (T).

The entry of nanoparticles into cells is a prerequisite for these biomedical applications. Receptor-mediated endocytosis has been supposed to be one of the main internalization pathways for nanoparticles to enter cells [7,8], during which the nanoparticle is adhered onto and wrapped by the cell membrane. Therefore, the wrapping behavior of a nanoparticle by the membrane is believed to be a key step of receptor-mediated endocytosis [9,10]. Recent studies indicate that the affinity between the receptors in the membrane and the ligands on the surface of a nanoparticle is one of the driving forces for the realization of membrane wrapping [11,12]. Moreover, the competition between such affinity and the deformation of the cell membrane further determines the cellular internalization process of the nanoparticle [13–15]. It is found by both experimental and theoretical investigations that the size, shape and surface chemistry of the nanoparticle have significant effects on both the formation of receptor-ligand binding and the membrane deformation, which consequently influence such an internalization process [13,16–22]. For example, the distinct geometrical characters of carbon nanotubes and graphene make their internalization processes completely different, although the chemical compositions of them are similar [13,16,23,24]. Under the influence of particle geometry on the internalization process of a nanoparticle, the rotation motion of the nanoparticle is observed [21,25]. Moreover, the orientation change in rotation of the nanoparticle is believed to further facilitate the internalization process [26]. In addition, the length, stiffness and coating pattern of ligands on the nanoparticle surface are found to possibly affect the wrapping manners of the nanoparticle by the membrane and the corresponding internalization pathways [27]. All these studies demonstrate the importance of the characteristics of nanoparticles, including ligands, on the proceeding of endocytosis. However, concerning the influence of the receptor’s structure and length, little is known. Furthermore, for the related computer simulations, different receptor models are applied, and even most simulations directly use the lipid model as that of the receptor [12,13,28,29]. However, the receptors involved in endocytosis are transmembrane proteins, whose size and structure greatly differ from those of lipid molecules [8]. Therefore, it is of great importance to study how the receptor structural factors influence the nanoparticle’s internalization process. In this paper, the influence of five types of receptors with different structures or lengths on the wrapping process of a nanoparticle by the membrane are investigated by using dissipative particle dynamics (Figure 1). We find that both the structure and length of the receptor affect the interaction configuration of the nanoparticle and the membrane and even the membrane states. These results are helpful to understand the nature of endocytosis and the related biomedical applications of nanoparticles.

## 2. Results and Discussion

### 2.1. Effect of Receptor Structure

We firstly concentrate on the influence of the number of the constituent chains of receptors (*N_m_*) on the membrane wrapping behavior of a nanoparticle. In order to avoid the aggregation of receptors in the lipid membrane caused by the hydrophobic mismatch effect, in this section, we set the length of the hydrophobic backbone of each constituent chain as *N_t_* = 6. Under this condition, the length of the hydrophobic part of a receptor is similar to the thickness of the sandwiched hydrophobic layer of the lipid membrane. Thus, the receptors distribute randomly in the whole membrane, and no obvious aggregation of the receptors is observed.

**Figure 2.**
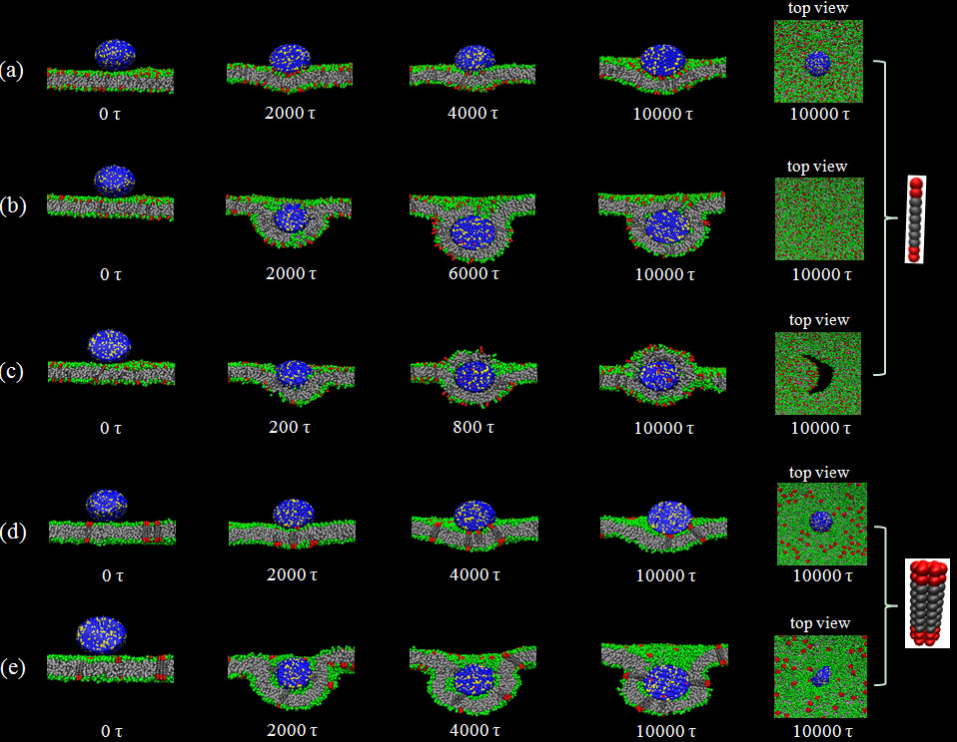
Different cases of the wrapping process of a nanoparticle (with ligands) by a lipid membrane with various embedded receptors. (**a**) Adhesion of the nanoparticle on the membrane. Receptor type: R1T_r_6; receptor number: *N* = 1120; ligand-receptor binding strength: ε = 3*k_B_T*; (**b**) wrapping of the nanoparticle by the membrane. Receptor type: R1T_r_6; receptor number: *N* = 1120; ε = 10*k_B_T*; (**c**) wrapping of the nanoparticle by the membrane along with the rupture of the membrane. Receptor type: R1T_r_6; receptor number: *N* = 1120; ε = 20*k_B_T*; (**d**) adhesion of the nanoparticle on the membrane. Receptor type: R7T_r_6; receptor number: *N* = 160; ε = 3*k_B_T*; (**e**) frustrated wrapping of the nanoparticle by the membrane. Receptor type: R7T_r_6; receptor number: *N* = 160; ε = 20*k_B_T*.

Figure 2a–c shows the wrapping process of a nanoparticle by a lipid membrane in which R1T_r_6-type receptors are embedded, under varying ligand-receptor affinity. Similar to the previous simulations [11–15,17], it is also found by our simulations that the membrane wrapping behavior is strongly influenced by the binding strength, ε, between the receptor and the ligand. When ε = 3*k*_B_*T*, the nanoparticle only adheres on the membrane surface at the end of the simulation (Figure 2a). However, with the increase of ε, the interaction configuration of the nanoparticle and the membrane changes dramatically: when ε = 10*k*_B_*T*, the nanoparticle is fully wrapped by the lipid membrane (Figure 2b); and when ε = 20*k*_B_*T*, the wrapping process is greatly accelerated. The strong nanoparticle-lipid membrane interplay even causes the rupture of the membrane (Figure 2c).

**Figure 3.**
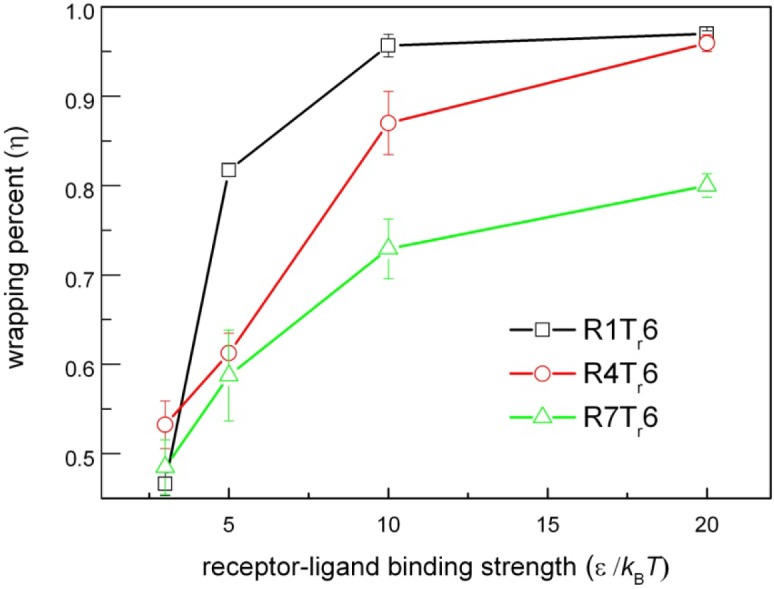
ε-η plots presenting the wrapping behaviors of nanoparticles by lipid membranes embedded with different types of receptors: R1T_r_6, R4T_r_6 and R7T_r_6.

**Figure 4.**
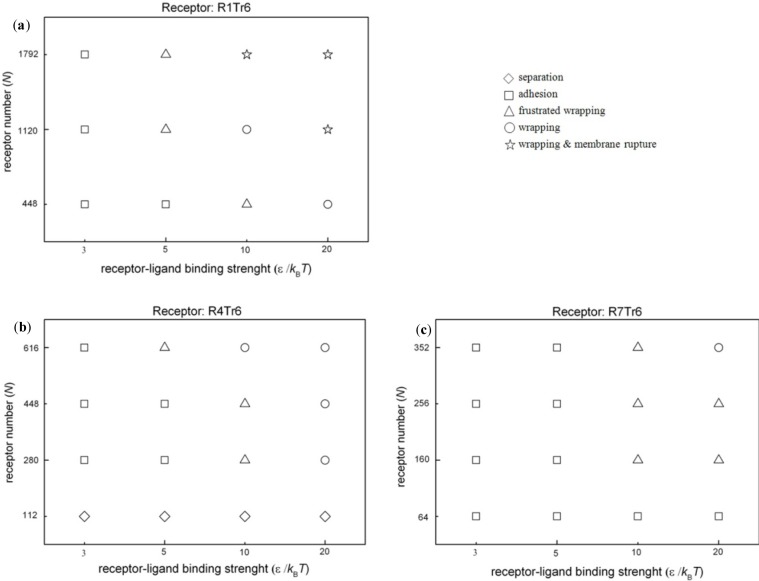
Interaction configurations of nanoparticles and lipid membranes embedded with three different types of receptors at the end of the simulations. (**a**) R1T_r_6; (**b**) R4T_r_6 and (**c**) R7T_r_6. ⋄: separation; □: adhesion (η < 0.7); Δ: frustrated wrapping (0.7 ≤ η ≤ 0.9); ○: wrapping (η > 0.9); ☆: wrapping and membrane rupture.

Interestingly, it is found that the structure of the receptor also affects the membrane wrapping behavior. When the type of the embedded receptor is changed from R1T_r_6 to R7T_r_6, we find that, even with the same number of active interaction sites of receptors in the membrane (*i.e.*, keeping the amount of the active beads of receptors in the membrane as the same as before), the interaction configurations of the nanoparticle and the lipid membrane change. Especially for the case of ε = 20*k*_B_*T*, the wrapping process of the nanoparticle by the membrane becomes rather slow. At the end of the simulation, the full wrapping has not been finished yet (Figure 2e). Such an influence is also reflected by the change of the wrapping percent, η, of the nanoparticle by the lipid membrane at the end of the simulations. Herein, η refers to the ratio between the area of the particle surface wrapped by the membrane and the total particle surface. As shown in Figure 3, with a fixed receptor-ligand binding strength, the receptor type indeed changes the membrane wrapping situation of the nanoparticle. Based on the calculation of η, the possible interaction configurations of nanoparticles and lipid membranes embedded with three types of receptors (*i.e.*, R1T_r_6, R4T_r_6 and R7T_r_6) at the end of the simulations are summarized in Figure 4. It is shown that when *N* (*i.e.*, the receptor number) is small or ε (*i.e.*, the ligand-receptor affinity) is low, the receptor-ligand binding cannot provide enough driving force for the membrane wrapping; thus, the nanoparticle is just adsorbed on, or even separated from, the surface of the membrane. With the increase of ε, it is possible for the wrapping to proceed, and the effect of the receptor structure also appears. It is shown that with the same possible active interaction sites between ligands and receptors, the proceeding of the wrapping behavior of the nanoparticle by the membrane becomes difficult with the increase of *N_m_*. According to the detailed interaction process between them (Figure 2), this change may be associated with the different distribution situations of the receptors around the nanoparticle.

### 2.2. Effect of Receptor Length

**Figure 5.**
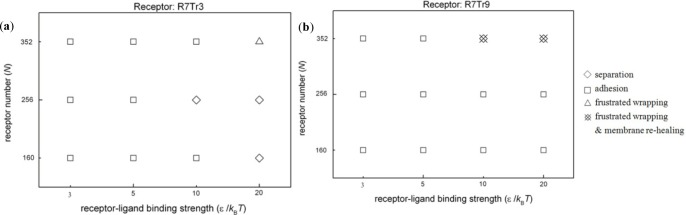
Interaction configurations between nanoparticles and lipid membranes embedded with two types of receptors, (**a**) R7T_r_3 and (**b**) R7T_r_9, under the case of hydrophobic mismatch, at the end of the simulations. ⋄: separation; □: adhesion (η < 0.7); Δ: frustrated wrapping (0.7 ≤ η ≤ 0.9); (Image007): frustrated wrapping and self-healing of the membrane after the rupture.

If the length of the hydrophobic part of the receptor does not match the thickness of the hydrophobic core of the lipid membrane, spontaneous aggregation of the receptors would occur under the effect of hydrophobic mismatch [30,31]. In this case, the membrane wrapping of nanoparticles becomes more complicated. Figure 5 shows the possible configurations of nanoparticles and lipid membranes embedded with different types of receptors, R7T_r_3 or R7T_r_9. Comparing with the cases mentioned in the above section, we find that the proceeding of membrane wrapping becomes more difficult. Full wrapping is hardly observed; instead, in most cases, the nanoparticle is only adhered on the membrane surface.

**Figure 6.**
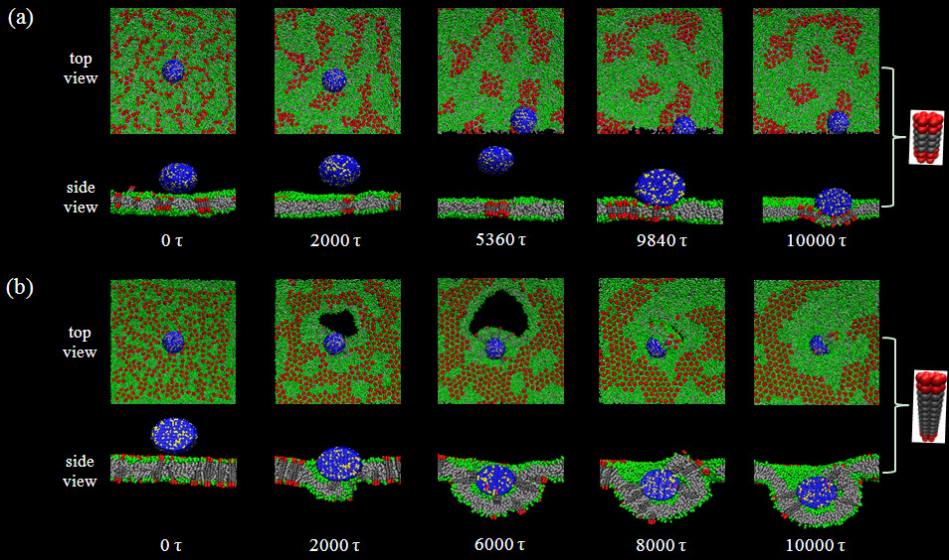
(**a**) Adhesion process of a nanoparticle on the surface of a membrane embedded with R7T_r_3-type receptors. *N* = 256; ε = 5*k_B_T*; (**b**) frustrated wrapping of a nanoparticle by a membrane embedded with R7T_r_9-type receptors and self-healing of the membrane after rupture. *N* = 352; ε = 10*k_B_T*.

Figure 6a shows a typical example of the adhesion process of a nanoparticle on a membrane surface with the existence of hydrophobic mismatch between the embedded receptors (R7T_r_3, *i.e.*, *N_t_* = 3) and the lipid membrane. Note that at the beginning, the receptors are randomly distributed in the membrane. Then, the aggregation of the receptors appears immediately, due to the hydrophobic mismatch effect. From Figure 6a, it is indicated that the influence of receptor aggregation on the nanoparticle-membrane interactions displays on two sides. On the one hand, such an aggregation may reduce the number of the receptors near the nanoparticle and, consequently, lower the possibility of binding between ligands and receptors. However, on the other hand, once the nanoparticle begins to contact the membrane, it will interact with not a single, but a cluster of aggregated receptors (Figure 6a). Despite this, the receptor cluster increases the local stiffness of the membrane around the adsorbed nanoparticle and, thus, tends to hinder the deformation of the lipid membrane. It is known that for the membrane wrapping behavior, the deformation of the membrane is absolutely necessary. Therefore, under the case of hydrophobic mismatch, the adhesion of a nanoparticle to a membrane is possibly blocked (e.g., the separation cases shown in Figure 5). Such a competition between the nanoparticle-membrane binding and membrane deformation plays key roles in determining the interaction configuration of nanoparticles and membranes. For the case shown in Figure 6b (R7T_r_9-type receptors, *N* = 352), although the nanoparticle is inclined to adhere on the membrane surface due to an increased receptor number and a stronger receptor-ligand binding strength, consequently, this leads to the local deformation of the membrane. However, because of the high rigidity of the aggregated receptors, the particle-membrane interaction also causes the rupture of the membrane and hinders the occurrence of full wrapping. Interestingly, with the proceeding of the wrapping process, the membrane can reheal spontaneously (Figure 6b). This self-healing of the lipid membrane may be related to the decrease of the membrane tension induced by the aggregation of receptors in the region, whose deformation degree is small during the wrapping process (Figure 6b).

## 3. Experimental Section

Dissipative particle dynamics (DPD) is a coarse-grained computer simulation technique [32]. Due to the application of “soft” potential, this technique can be used to simulate a complex system, such as lipid membranes [12,29,33,34]. The main interaction between the beads is denoted as

$${\vec{F}}^{C}{}_{ij}=a_{ij}(1-\frac{r_{ij}}{r_{c}}){\vec{e}}_{ij}$$

where ${\vec{r}}_{ij}={\vec{r}}_{i}-{\vec{r}}_{j},\;{\vec{e}}_{ij}={\vec{r}}_{ij}/r_{ij},\;r_{c}$ is the cut-off radius of the force and *a_ij_* is the maximum repulsion interaction of beads of Type *i* and Type *j*. In additional, dissipative and random forces are applied to each pair of neighboring beads to keep the momentum locally conserved and produce the hydrodynamic effect. In the simulations, a modified velocity-Verlet integration algorithm [32] is applied, and the integration time step is Δ*t* = 0.02τ [τ = (*mr*_c_^2^/*k*_B_*T*)^1/2^].

The lipid molecule used in the simulations is modeled as a linear chain with two hydrophilic head beads (H) and five hydrophobic tail beads (*T*). To connect the neighboring beads in a single molecule, a harmonic spring potential, *U*_s_ (= $k_{s}{\left(1-\frac{r_{i,i+1}}{l_{0}}\right)}^{2},$ where *k*_s_ = 128*k*_B_*T/r*_c_ and *l*_0_ = 0.5*r*_c_), is used. Furthermore, a three-body bond angle potential *U_b_* (*U*_b_ = *k*_bd_(1 – cos(φ – φ_0_)) where *k*_bd_ = 5*k*_B_*T/r*_c_ and φ = 0 is applied to ensure the rigidity of lipid tails. It has been proven that this model could well reproduce the phase behaviors of lipid molecules [35,36]. In our simulations, these lipids can self-assemble into a bilayer membrane with a thickness of 5*r*_c_ (the thickness of the hydrophobic core of the membrane is 3*r*_c_, Figure 1). The bending modulus of the bilayer is about 13*k*_B_*T*, which is in an experimentally interesting range [37].

In the simulations, a receptor consists of *N_m_* polymer chains with equal length (as shown in Figure 1). Each of such a chain has *N_t_* hydrophobic beads (T_r_) as the backbone, and at the two ends of the chain, two hydrophilic beads (H_r_) are attached as the active interaction sites of the receptor, respectively. For the integrity of the receptor, the neighboring beads in each chain are connected by the spring potential, *U*_s_. Furthermore, these beads are linked to the corresponding beads in the neighboring chains by the same spring potential, *U*_s_. Thus, the receptor used in the simulations is actually a bundle of *N_m_* of these amphiphilic chains. Furthermore, the three-body bond angle potential, *U*_b_, is also applied to the beads in each chain. To ensure the rigidity of the receptor, *k*_b_ is increased to 80*k*_B_*T/r*_c_. According to the number of the constituent chain (*N_m_*) and the number of the hydrophobic beads in each chain (*N_t_*), the receptor used in our simulations can be characterized as R1T_r_6 (*N_m_* = 1, *N_t_* = 6), R4T_r_6 (*N_m_* = 4, *N_t_* = 6), R7T_r_6 (*N_m_* = 7, *N_t_* = 6), R7T_r_3 (*N_m_* = 7, *N_t_* = 3), R7T_r_9 (*N_m_* = 7, *N_t_* = 9), and so on.

The nanoparticle used in the simulation is fabricated by arranging the hydrophilic beads (*P*) on an fcc lattice into a spherical shape [38]. Due to the close packing of beads in the nanoparticle, water or other beads cannot enter the interior of the nanoparticle. All beads comprising a nanoparticle move as a rigid body. Based on the previous simulations [13,17,21], about 50% of the surface beads of the nanoparticle are randomly chosen as the ligands (L), namely the ligand beads are randomly distributed on the hydrophilic surface of the nanoparticle. Additionally, water is explicitly included in the system as the solvent (W).

According to the hydrophobic/hydrophilic characteristics of beads, the interaction parameters set is *a_ij_* = 25*k*_B_*T/r*_c_ for the beads with the same types and *a_ij_* = 95*k*_B_*T/r*_c_ for different types. There are 7 types of beads in our simulations. Among them, W-, H-, H_r_-, P- and L-type beads are hydrophilic, but T- and T_r_-type beads are hydrophobic. Additionally, to mimic the specific receptor-ligand interaction, a modified Lennard-Jones potential [39],

$$U_{ij}=4\varepsilon [{\left(\frac{\sigma }{r_{ij}}\right)}^{12}-{\left(\frac{\sigma }{r_{ij}}\right)}^{6}]+0.22\varepsilon$$

with σ = 0.62*r*_c_, is applied between the ligand (L-type bead) and the active interaction site of the receptor (H_r_-type bead), in addition to the soft repulsive force in the DPD technique. This potential will be truncated if the corresponding repulsive force is larger than 10*k*_B_*T/r*_c_ to guarantee the proper running of the DPD simulation. The value of σ and the factor, 0.22ε, make sure *U_ij_* will be truncated at *r*_c_, as other non-bonded potentials in DPD.

In order to obtain the initial configuration of the simulation system, the receptors are firstly included into a pre-equilibrated planar lipid bilayer membrane randomly. Moreover, a number of lipids are removed to keep the zero surface tension of the membrane. This membrane with the embedded receptors is then placed in the center of the simulation box (*x*-*y* plane; the simulation box’s size is 64*r*_c_ × 64*r*_c_ × 48*r*_c_). A nanoparticle is placed close to the membrane surface (about *r*_c_). Then, the system is equilibrated, while the location of nanoparticle remains unchanged (for the receptors with a hydrophobic mismatch, their locations are also fixed during the equilibrium), which is applied as the initial configuration of the simulation. Furthermore, to maintain the tensionless state of the membrane in the whole simulation, the number of lipids in the boundary (the width is 4*r*_c_) of a bilayer membrane can be added or deleted accordingly. It has been proven that this method is very suitable for the studies of the deformation behaviors of lipid membranes [12,26,34]. The simulations are performed in isochoric and isothermal ensembles with periodic boundary conditions at the temperature *k*_B_*T*= ε. All simulations are carried out at least 5 × 10^5^ simulation steps (10,000τ) and 3 independent runs.

## 4. Conclusions

In summary, the effects of the length and structure of receptors on the wrapping behavior of a nanoparticle by the membrane is investigated by computer simulations. It is found that, with various types of receptors, the interaction configurations of the nanoparticle and the membrane could be separation, adhesion and full or part wrapping, possibly with the rupture of the membrane. Additionally, the occurrence of these behaviors are tightly associated with the differences in the length of the receptor and the number of chains composing the receptor. It is shown by our simulations that for different types of receptors, their distribution in the membrane is different, which consequently affects the local stiffness of the membrane. These factors further influence the interaction between the nanoparticle and the lipid membrane, as well as the deformation of the membrane.

It should be noted that our simulation models of the receptor and even the lipid bilayer have differences with the real cell membrane system. Our purpose is, with the aid of computer simulations, to help people to better understand the significance of the physical properties of the receptor on the cellular internalization process, which was overlooked before. Additionally, our results also provide useful hints for the design of nanoscale cargo carriers to cells: if the effect of the physical properties of receptors on the nanoparticle-membrane interaction can be fully considered in the preparation of nanoparticles, the internalization pathways of the nanoparticles are possibly controlled.
